# Bacterial Cellulose-Hydroxyapatite Nanocomposites for Bone Regeneration

**DOI:** 10.1155/2011/175362

**Published:** 2011-09-27

**Authors:** S. Saska, H. S. Barud, A. M. M. Gaspar, R. Marchetto, S. J. L. Ribeiro, Y. Messaddeq

**Affiliations:** ^1^Institute of Chemistry, University Estadual Paulista—UNESP, CP 355, 14-801-970 Araraquara, SP, Brazil; ^2^Department of Inorganic Chemistry, Institute of Chemistry—UNESP, Rua Francisco Degni s/n, 14-800-900 Araraquara, SP, Brazil; ^3^Department of Morphology, Dental School, University Estadual Paulista—UNESP, Rua Humaitá, 1680, 14-801-903 Araraquara, SP, Brazil

## Abstract

The aim of this study was to develop and to evaluate the biological properties of bacterial cellulose-hydroxyapatite (BC-HA) nanocomposite membranes for bone regeneration. Nanocomposites were prepared from bacterial cellulose membranes sequentially incubated in solutions of CaCl_2_ followed by Na_2_HPO_4_. BC-HA membranes were evaluated in noncritical bone defects in rat tibiae at 1, 4, and 16 weeks. Thermogravimetric analyses showed that the amount of the mineral phase was 40%–50% of the total weight. Spectroscopy, electronic microscopy/energy dispersive X-ray analyses, and X-ray diffraction showed formation of HA crystals on BC nanofibres. Low crystallinity HA crystals presented Ca/P a molar ratio of 1.5 (calcium-deficient HA), similar to physiological bone. Fourier transformed infrared spectroscopy analysis showed bands assigned to phosphate and carbonate ions. *In vivo* tests showed no inflammatory reaction after 1 week. After 4 weeks, defects were observed to be completely filled in by new bone tissue. The BC-HA membranes were effective for bone regeneration.

## 1. Introduction

Recently, a new generation of resorbable materials has been developed for soft and/or bone tissue regeneration purposes [1–4], including bacterial cellulose (BC), which has shown possible osteoconduction properties [5–10]. 

BC is obtained from cultures of a Gram-negative bacteria, *Gluconacetobacter xylinus, *which produces highly hydrated membranes (up to 99% water), free of lignin and hemicelluloses, and which presents a higher molecular weight and crystallinity than plant cellulose. BC membranes show great elasticity, high wet strength, and conformability [11]. The gelatinous membrane formed in static culture is characterized by a 3D structure consisting of an ultrafine network of cellulose nanofibres (“nanocelluloses”), resulting in a large nanoporous surface area. The unique properties provided by the nanometric structure have led to a number of commercial products and medical applications such as wound dressings and skin substitutes [12]. BC membranes present important characteristics such as biocompatibility, bioinertness, biodegradability, and selective permeability. Furthermore, they also play the role of barrier against microorganisms in wounds and burns, accelerating the healing process, providing pain relief and reducing scar formation [13–15]. BC membranes have been used for guided bone regeneration (GBR) in bone defects of critical and noncritical size, in periodontal lesions, and as a resorbable barrier membrane occluding fibroblastic cells and fibrous connective tissue into bone defects. Moreover, the results from the literature have revealed that BC membranes promote effective bone formation at the site, besides being a low-cost treatment [16–19]. Fang et al. [20] showed that HA/BC nanocomposite scaffolds developed were biocompatible and could promote cell proliferation and differentiation *in vitro* using stromal cells derived from human bone marrow (hBMSC). Other *in vitro* studies have revealed that BC-HA composites have a great potential for application in tissue engineering or bone regeneration as well [21, 22]. Therefore, BC-hydroxyapatite (BC-HA) composites should also be good candidates for bone regeneration, because this composite may promote better bone regeneration, mainly for GBR. Basically, two main routes have been proposed for the preparation of composite membranes. The first includes a biomimetic route in which the BC membrane is submitted to pretreatment with a Ca(OH)_2_ or CaCl_2_ solution. Afterwards, the membrane is soaked in an SBF solution, where HA formation occurs mainly at the membrane surface [5, 6, 8–10]. The second route includes cyclic treatments with CaCl_2_ and sodium phosphate solutions [7]. 

This paper aims to describe the preparation and characterization of BC-HA composite membranes; in addition, biological behavior of the composite membranes was evaluated in noncritical bone defects in rat tibiae.

## 2. Materials and Methods

### 2.1. Preparation of Bacterial Cellulose-Hydroxyapatite Composites (BC-HA)

Bacterial cellulose membranes were supplied from Fibrocel (Produtos Biotecnológicos LTDA, Ibiporã, PR, Brazil).

Incorporation of hydroxyapatite (HA) into the BC hydrogel was performed following the methodology proposed by Hutchens et al. [7]. Highly hydrated BC membranes (2 × 2 cm^2^, 5 mm thickness) were immersed in 20% ethanol at room temperature (25°C) for 24 h. HA was formed in BC by alternating incubation cycles in 20 mL of 0.05 mol·L^−1^ CaCl_2_ solution (pH 5.8) and 20 mL of 0.1 mol·L^−1^ Na_2_HPO_4_ solution (pH 9.1) at 25°C. The samples were dried at 50°C for 7 days and sterilized by gamma radiation (20 kGy).

### 2.2. Characterization of BC-HA Composites

Thermogravimetric (TG) curves of the dried samples were recorded using a TA SDT 2960 from TA Instruments Co. Samples were heated in open *α*-alumina pans from 40°C to 600°C under a nitrogen atmosphere (flow rate: 70 mL min^−1^) at a heating rate of 10°C min^−1^. X-rays diffraction (XRD) patterns were obtained using a Kristalloflex Simens Diffractometer with a Ni filter and Cu K*α* radiation from 4° to 70°. Fourier transform infrared (FT-IR) spectra were obtained from dried powdered samples on a Perkin Elmer Spectrum 2000 Fourier transform infrared spectrophotometer. Pellets were prepared from mixtures of the samples and KBr (1 : 100 in weight). Thirty-two scans were accumulated at a resolution of 4 cm^−1^. Scanning electron microscopy (SEM) images and energy-dispersive X-ray spectroscopy (EDS) analysis were obtained from an FEG XL 30-Philips. After EDS analysis, samples were coated with a 1 nm thin layer of gold for 60 s (3 kV and 9.5 *μ*A).

### 2.3. *In Vivo* Experiment

Eighteen male adult rats (*Rattus Norvevicus Holtzman*) were used in the study. The Animal Experimentation Ethics Committee of the Pharmaceutical School of Araraquara-UNESP approved all animal experimental protocols. General anesthesia was induced using intramuscular injections of ketamine hydrochloride (0.1 mL/100 g; Agener União, Brazil) and xylazine hydrochloride (0.01 mL/100 g; Bayer, Brazil). Surgery was performed using standard aseptic techniques. An incision of approximately 10 mm was made on the anterior region of each tibia. Tibiae were exposed and one noncritical size bone defect (2 mm in diameter) approximately 1 cm from the distal extremity was created in each tibia based on a previous study [23]. The bone defects were performed with a surgical drill (2 mm in diameter; Neodent, Brazil), under copious saline irrigation, drilling into the full thickness of cortical and exposing the bone marrow. In the treated group, the left tibia defects were filled with a blood clot and recovered by the BC-HA barrier composite membrane (4 mm × 4 mm). In the control group, the right tibia defects were filled with a blood clot only. The flaps were sutured with 4–0 mononylon (Ethicon, Johnson & Johnson, Brazil). In the immediate postoperative period all animals received an oral administration (single dose) of 120–300 mg/kg of salicylic acid (Bayer-Brazil). Six animals were evaluated after 1, 4, and 16 weeks. The specimens were reduced, preserving the periosteum. The samples were fixed in Bouin for 72 hours before further analysis. Decalcification of the samples was performed with a solution containing equal parts of 50% formic acid and 20% sodium citrate. Routine histological processing for light microscopy was carried out, and 6 *μ*m sections were stained with hematoxylin-eosin (H. E.). Sections were analyzed under an optical microscope (Jenaval-Zeiss) coupled to a digital camera (Leica DFC425). The parameters analyzed were examined by a researcher who was blinded to the two treatment groups for mineralized bone quality, cellular activity including osteoblasts, osteoids, and osteocytes as well as any evidence of fibrotic tissue formation within the defect site, angiogenesis processes, inflammatory reactions, and degradation of the membranes. The results of degradation of the BC-HA membranes were analyzed using BioEstast 5.0 statistical software. A two-way ANOVA was carried out to evaluate the measured values (ImageJ software) from membranes for statistical significance among periods 7, 30, and 120 days. Statistical significance was established at *P* < 0.05.

## 3. Results and Discussion

### 3.1. Characterization of BC-HA Nanocomposites

**Figure 1:**
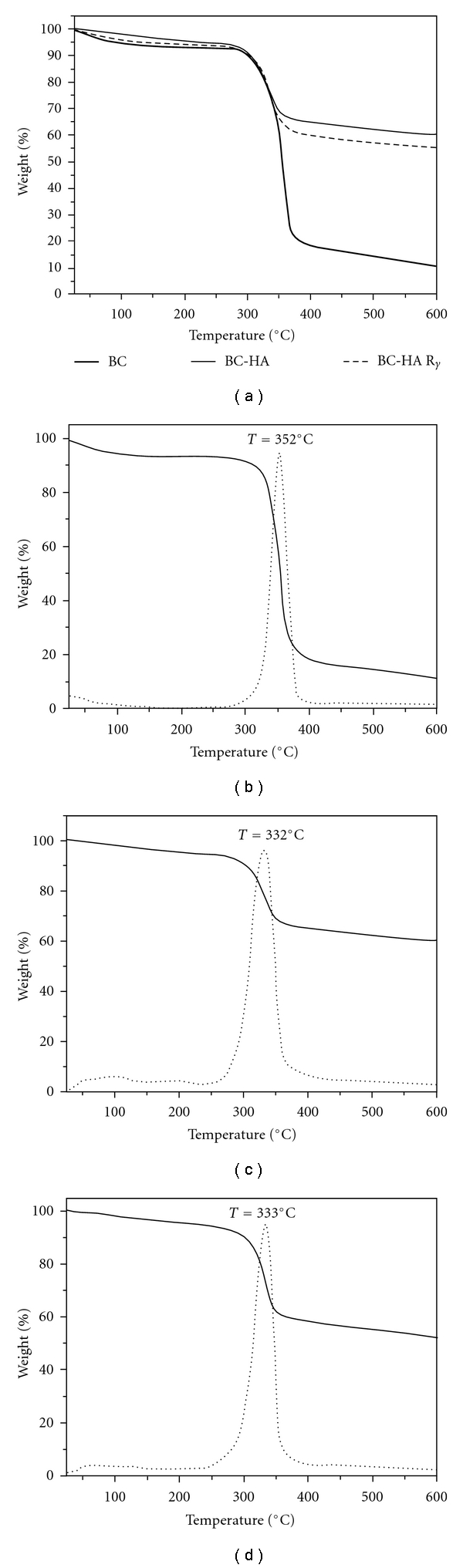
(a) TG curves: (thick line) bacterial cellulose (BC), (solid line) BC-HA and (dashed line) BC-HA sterilized by 20 kGy gamma radiation (BC-HA*γ*); TG curve (solid line) and DTG curve (dashed line) of BC (b), BC-HA (c) and BC-HA*γ* (d).

Figure 1 shows the TG results of the BC and BC-HA composite membranes with or without gamma radiation sterilization. Thermogravimetric analysis was carried out to estimate thermal stability and degradation profiles of the BC and BC-HA composites.

The samples showed an initial smooth weight loss from ambient temperature up to 230°C (5%–10%) due to water and solvent loss [5, 24].

At around 320°C–350°C decomposition of the samples led to an important weight loss. These events could be associated with a cellulose degradation process including depolymerization, dehydration, and decomposition of glucosyl units followed by the formation of a charred residue [5, 24, 25].

A carbonaceous residue was observed for the pure BC membrane, around 10% at 600°C. BC-HA composites presented a residue around 60%, confirming HA deposition on the BC membrane, which means that HA content was around 50%. 

The onset temperature (*T*_onset_) observed in the TG curves revealed that the thermal stability of BC decreased with the presence of HA. The (*T*_onset_) of BC was at 352°C, and (*T*_onset_) of the composites, BC-HA and sterilized BC-HA, were at 332°C and 333°C, respectively. This behavior may be associated with broken hydrogen bonds and the reduced crystallinity of BC; thus, reduced crystallinity leads to a decrease in (*T*_onset_) values [26]. According to Gao et al. [26], HA crystals did not show sufficient barrier properties for delaying heat and gas diffusion to BC, because HA nanoparticles did not cover the entire surface of BC nanofibres [26]. Furthermore, gamma radiation did not promote changes in the characteristic temperatures of BC-HA composites. This result permitted us to infer that gamma radiation is an adequate treatment for BC-HA composite sterilization.

**Figure 2:**
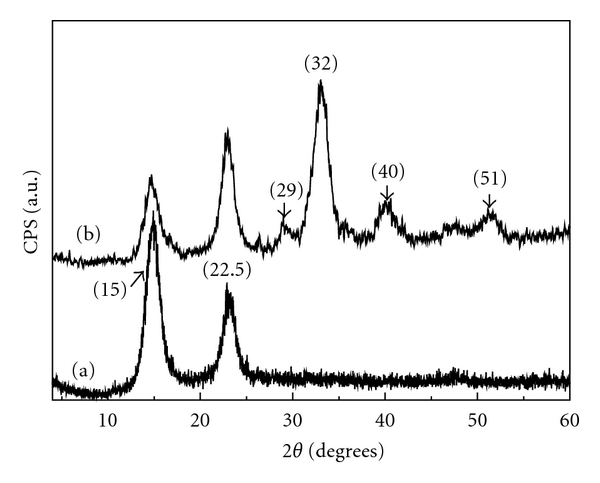
X-ray diffraction patterns of the BC (a) and BC-HA nanocomposites (b). The characteristic HA peaks are indexed at the top for BC-HA.

Figure 2 shows the X-ray diffraction patterns obtained for the BC membrane and BC-HA composite. Typical BC crystalline phases were observed in both samples. Characteristic peaks of BC-HA crystals were identified as JCPDF 46-0905. Diffraction peaks at 2**θ** = 15° and 22.5° were assigned to the cellulose I*α* and I*β* phases (100_1*α*_, 110_1*β*_ and 010_1*β*_ planes at 15° and 110_1*α*_ and 200_1*β*_ at 22.5°) [27]. The main characteristic diffraction peaks of HA crystal phase were observed at 2**θ** = 29°, 32°, 40°, and 51° (Figure 2(b)). XRD pattern was in fact very similar to the XRD pattern of bone apatite, suggesting low crystallinity [28]. According to Hutchens et al. [7], the decrease in the intensity of the cellulose peaks in comparison with the pattern obtained from the pure BC membrane was due to HA deposition on BC nanofibrils. The residue observed in thermogravimetric analysis for temperatures above that of cellulose decomposition averaged 50% and must be related to the HA weight average.

**Figure 3:**
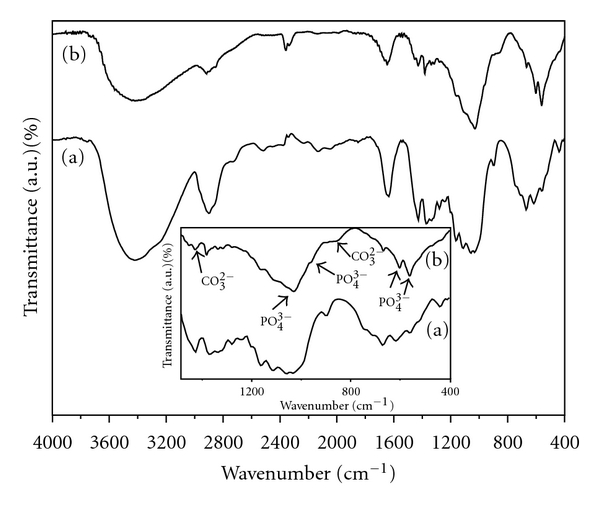
FTIR spectra of the BC (a) and BC-HA nanocomposites (b). Characteristic hydroxyapatite bands correspond to PO_4_^3−^ and CO_2_^3−^ ions (inset figure).

The FT-IR spectra of dried BC membranes and BC-HA composite membranes are shown in Figure 3. Characteristic vibrational frequencies assigned to cellulose were observed at 3500–3200 cm^−1^ (OH stretching), 2908 cm^−1^ (CH stretching of CH_2_ and CH_3_ groups), 2700 cm^−1^ (CH_2_), 1645 cm^−1^ (water OH bending), 1435 cm^−1^ (CH_2_ symmetric bending), 1370 cm^−1^ (CH bending), 1160 cm^−1^ (antisymmetric bridge C–O–C stretching), 1111 cm^−1^, and 1056 cm^−1^ (skeletal vibrations involving C–O stretching) [27]. The band in the region from 3500–3200 cm^−1^, assigned to cellulose hydroxyl groups, was observed with decreasing relative intensity for the composite in comparison with the pure BC membrane. This decrease in intensity suggests that the presence of the HA crystals affected the cellulose hydroxyl groups. Moreover the red shift observed for the band assigned to intramolecular hydrogen bonding (~3500 cm^−1^) confirms strong interaction between the OH group and apatite. The chemical interaction between HA and BC stabilizes the composite so that it can maintain the mechanical integrity necessary for bone substitution. FT-IR bands observed for BC-HA composite at 1093, 1020, 962, and 570–600 cm^−1^ were attributed to vibrational modes of PO_4_^3−^ ions [5–8]. The 962 cm^−1^ peak showed up as a shoulder of the stronger band at the 1020 cm^−1^ peak, and the weak bands at 1418 and 838 cm^−1^ (inset picture) correspond to the stretching mode of CO_3_^2−^ ions, suggesting absorption of CO_2_ from the air. The presence of the PO_4_^3−^ doublet band at 602 cm^−1^ and 567 cm^−1^ (inset figure) strongly suggest that the precursor phase of the HA was the OCP [29]. Therefore, FT-IR analysis suggested carbonate-containing apatite (bonelike apatite) deposited on BC nanofibres.

**Figure 4:**
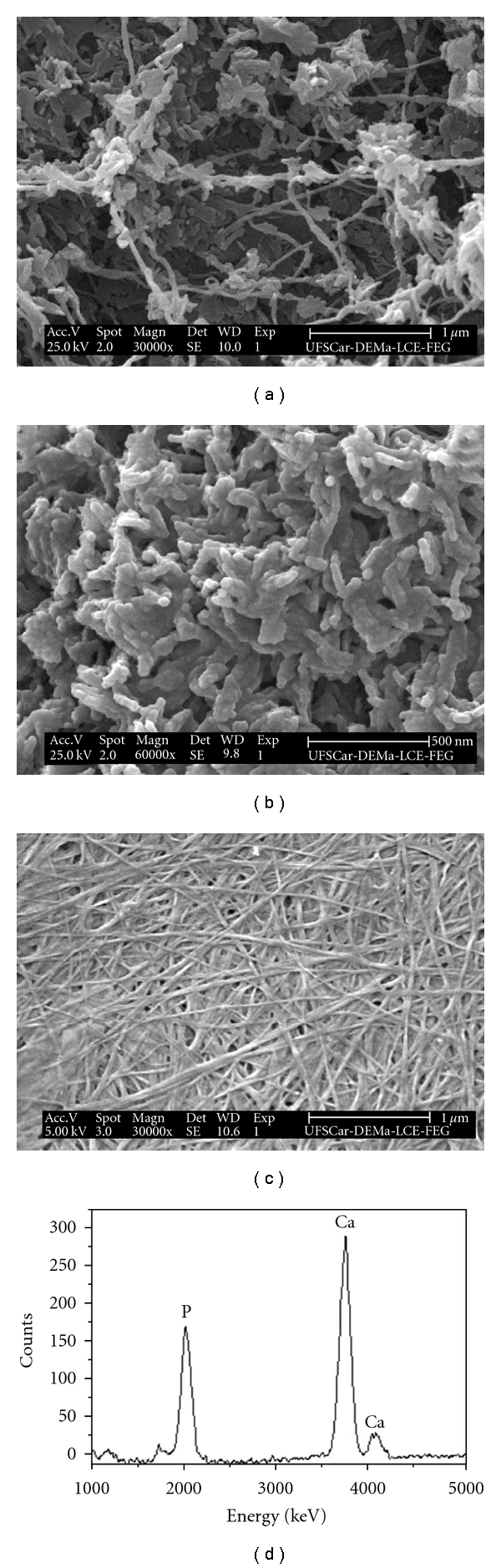
SEM images of the BC-HA nanocomposites (a) and (b) at 30.000× and 60.000×, respectively, and SEM images of the BC membrane (c) at 30.000×. A 5 kV accelerating voltage was used for the BC sample, and a 25 kV voltage was used to obtain the image of the BC-HA nanocomposites. EDS spectrum was taken from a typical nanofibril with surrounding HA crystals (d).

SEM images of the BC and BC-HA composites are shown in Figure 4.  Figure 4(c) shows a typical SEM image of dried BC membrane. An ultrafine network structure formed by continuous nanofibres about 10–50 nm wide (“nanocelluloses”) can be observed. This nanometric structure leads to a large surface area for particle stabilization [30]. Figures 4(a) and 4(b) show SEM images of the dried BC-HA nanocomposite. HA nanocrystals were precipitated on the BC nanofibrils as agglomerates of crystallites Figure 4(a); regularly distributed pores were observed at the membrane surface. Typical HA crystals in globular and rod form were observed, similar to the HA crystals as has also been observed by other authors [31, 32]. These crystals grew laterally around the nanofibres. According to EDS analysis, the Ca/P molar ratio of the BC-HA composites was 1.50 suggesting calcium-deficient HA (CDHA), which is a phase similar to biological apatite. The low crystallinity was consistent with the broadness of the diffraction peaks of HA shown in Figure 2(b). Moreover, this Ca/P molar ratio favors faster dissolution of the Ca^2+^ and PO_4_^3−^ ions. Lower Ca/P molar ratio values lead to higher dissolution of Ca^2+^ ions, with an increase in local pH at the biomaterial/tissue interface, promoting the ideal pH for alkaline phosphatase activity. Therefore, osteoblast proliferation and synthesis of bone matrix may be increased. 

Calcium phosphates have different solubility and the comparative extent of dissolution is: OCP (Ca/P = 1.33) ≫ ß-tricalcium phosphate (ß-TCP) (Ca/P = 1.48) > calcium-deficient HA (CDHA) (Ca/P = 1.5) ≫ HA (Ca/P = 1.67). This difference reproduces the influence of composition and crystallographic properties of calcium phosphate, since stoichiometric HA is insoluble in body fluids [33, 34].

### 3.2. Histological Analysis

**Figure 5:**
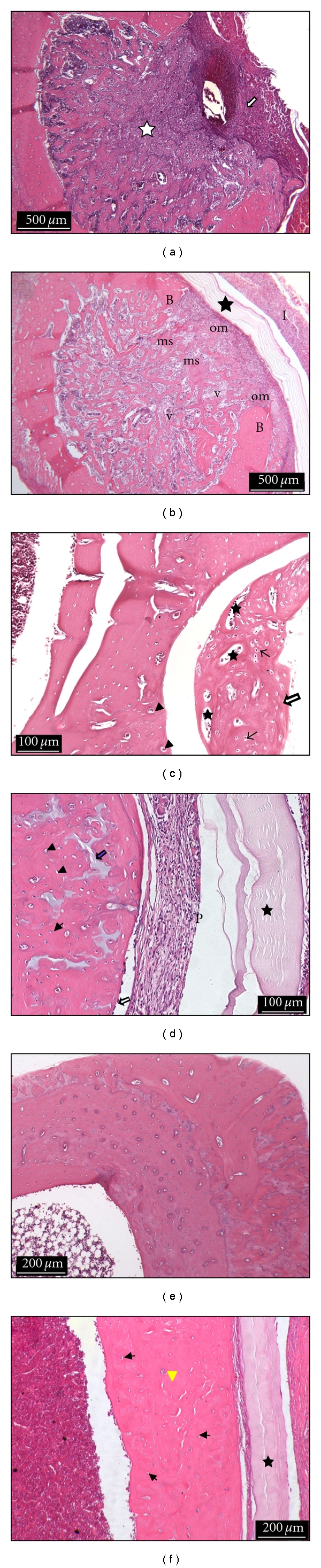
Histological photomicrographs: (a) Control group: 1 week. Bone defect filled by fibrotic tissue (white star); inflammatory infiltrate (white arrow). Hematoxylin-eosin staining (HE), scale bar (500 *μ*m); (b) Treated group: 1 week. Bone defect filled by newly formed bone, osteoids (om), medullary spaces with mesenchymal cells (ms), and several blood vessels (v); mature bone (B); BC-HA membrane (black star); inflammatory infiltrate (I) (HE), scale bar (500 *μ*m); (c) Control group: 4 weeks. New formed bone tissue is observed with several osteocytes (black arrows), blood vessels (arrow heads), and medullary spaces (stars); bone defect is not filled completely (white star) (HE), scale bar (100 *μ*m); (d) Treated group: 4 weeks. BC-HA membrane (star), periosteum (P), osteoblasts (white arrow), osteocytes (arrow heads), and bone matrix (blue arrow) (HE), scale bar (100 *μ*m); (e) Control group: 16 weeks. Mature bone (HE), scale bar (200 *μ*m). (f) Treated group: 16 weeks. BC-HA membrane (star); bone defect completely repaired by mature bone, osteocytes (black arrows), and blood vessels (arrow head) (HE), scale bar (200 *μ*m).

Figures 5(a) and 5(b) show images obtained from the control and treated groups after 1 week, respectively. The BC-HA nanocomposite membrane, osteoblasts, osteoids, newly formed bone, and medullary spaces with mesenchymal cells were observed within the defect site. However, in the control group, fibrotic tissue formation was observed within the defect site. Moderate inflammatory reaction could be observed for both groups in this period. At 4 weeks, newly formed bone tissue containing medullary spaces with mesenchymal cells, several osteocytes, and blood vessels were observed for control group; however, the newly formed bone was not integrated with tibia bone as shown in Figure 5(c).  Figure 5(d) shows that after 4 weeks, bone defects were observed to be filled by new formed bone with several osteocytes, blood vessels, and bone matrix in process of mineralization; the BC-HA membrane was observed in this period. Inflammatory reaction was not found in these two groups at 4 weeks. After 16 weeks, the BC-HA membrane was observed yet. Bone defects were completely repaired by mature bone for both groups (Figures 5(e) and 5(f)). 

BC-HA nanocomposite membranes were biocompatible and did not promote an inflammatory reaction after 4 weeks. Literature shows, in fact, that BC derivatives alone have been employed in soft tissues showing no inflammatory or foreign body reaction [15, 35]. It must be mentioned that HA-plant cellulose derivatives have been employed in rat bone defects showing inflammatory and foreign body reactions. In another experiment, plant cellulose sponges were filled by connective tissue and did not promote complete bone defect ossification [36].

Promising results have been obtained using BC membranes in intrabony periodontal defects as low-cost resorbable barriers [16, 17]. Clinical results were observed to be similar to those obtained from more expensive e-PTFE barriers in intrabony periodontal defects promoting effective new bone formation [16]. Studies have demonstrated that there was no additional advantage of using associated other alloplastic materials compared with BC membrane alone [18, 19]. 

In the present study, no membrane exposure was observed. Similar results were observed by dos Anjos et al. [16] and by Simonpietri et al. [19]. However, Batista et al. [18] observed membrane exposure using BC membranes in guided bone regeneration at a rate of 15.38% in the first 10 days; however, no abscesses or acute inflammatory reaction could be observed. 

Bone defects treated with BC-HA nanocomposite membranes presented defects that were filled by newly formed bone tissue organized and incorporated to the tibia bone in a period of 4 weeks. In the control group, new formed bone tissue was observed to be partially incorporated to the tibia bone in the same period of time. After 16 weeks, no differences could be observed between the groups. Bone defects were completely filled by mature bone for both groups. 

There are few studies reported in the literature evaluating the biological properties of BC-HA composites *in vitro* [20–22]. Moreover, there are no reports in the literature studying BC-HA composites in *in vivo* studies. However, there are several studies in the literature evaluating the biological properties of BC membranes in *in vivo* [13–19, 35, 37, 38], whose studies have revealed that BC membranes are a biomaterial in potential for application in tissue regeneration due to their great biological properties. Thus, the results of this study are pioneering in that they showed that BC-HA nanocomposites were compatible with the examined structures, including hard and soft tissues.

The ANOVA showed that there was a significant statistical difference regarding the degradation of BC-HA membranes between 7 and 120 days (*P* < 0.05). In addition, the effect of degradation of the membranes depend on the period analyzed (*P* = 0.03). The mean values of the degradation of the membranes are showed in Table 1. Mello et al. [37] observed similar results for reabsorption of the BC membranes as substitute for the dura mater in dogs, and decrease in membranes thickness was statistically significant between 30 to 270 days.

Biomaterial reabsorption is related to several factors such as particle size, porosity, chemical structure (composition and crystallinity), and pH of body fluids [34]. Particles with nanometric sizes are reabsorbed faster than micrometric particles, because osteoclasts or macrophages act more readily on a biomaterial surface. Biomaterial crystallinity also changes the reabsorption rate, since highly crystalline structures are more resistant to reabsorption than an amorphous or semicrystalline structure. Moreover, the chemical composition is also important. Impurities such as calcium carbonate promote faster reabsorption. Therefore, the chemical structure of BC-HA nanocomposite membranes, the HA particle, and BC nanofibres sizes suggests favoring reabsorption of this new biomaterial.

## 4. Conclusion

BC-HA composites have presented HA nanocrystals of low crystallinity in the membranes with a Ca/P molar rate similar to that of physiological bone. The BC-HA membranes were effective for bone regeneration in bone defects of rat tibiae, since the membranes accelerated new bone formation at the defect sites; in addition, reabsorption of the membranes was slow, suggesting that this composite takes longer to be completely reabsorbed.

## Figures and Tables

**Table 1:** Mean of the values of the reabsorption of the BC-HA membranes and standard deviations (different letters indicate statistical difference among periods according Turkey's test (*P* < 0.05)).

	Mean (mm)	Standard deviations (mm)	
7 days	0.18	0.03	A
30 days	0.17	0.02	A, B
120 days	0.13	0.03	B
